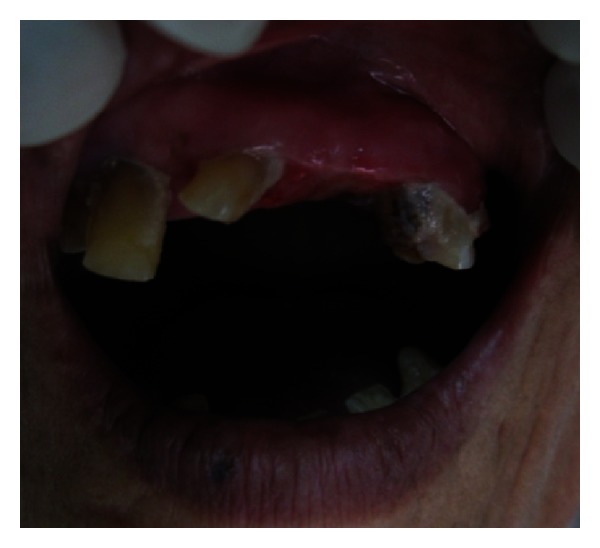# Peripheral Ossifying Fibroma

**DOI:** 10.1155/2013/497234

**Published:** 2013-05-20

**Authors:** Meenakshi Bhasin, Vinny Bhasin, Abhilasha Bhasin

**Affiliations:** ^1^Department of Oral Medicine, Mansarovar Dental College, Bhopal, Madhya Pradesh 462042, India; ^2^Department of Oral Medicine & Radiology, 153 Adarsh Nagar, Narmada Road, Jabalpur, Madhya Pradesh 482002, India; ^3^Department of Orthodontia, Mansarovar Dental College, Bhopal, Madhya Pradesh 462042, India; ^4^Department of Prosthodontics, Hithkarni Dental College, Jabalpur, Madhya Pradesh 482002, India

## Abstract

Intraoral ossifying fibromas have been described in the literature since the late 1940s. Peripheral ossifying fibroma (POF) is usually a fibroma of the gingival which shows areas of calcification or ossification. It is a nonneoplastic enlargement of gingiva. Due to its clinical and histopathological similarities, some POFs are believed to develop initially as a pyogenic granuloma that undergoes fibrous maturation and subsequent calcification. It has been suggested that POF represents a separate clinical entity rather than a transitional form of pyogenic granuloma or irritation fibroma. This paper describes a case report of a 60-year-old female patient reported with growth on gingiva in the upper left front region of mouth three years ago.

## 1. Introduction 

Many types of localized reactive lesions are seen on the gingiva, including focal fibrous hyperplasia, pyogenic granuloma, peripheral giant cell granuloma, and peripheral ossifying fibroma (POF) [1–3]. Synonyms of POF are peripheral cementifying fibroma, calcifying or ossifying fibroid epulis, and peripheral fibroma with calcification. These lesions may arise as a result of irritants such as trauma, microorganisms, plaque, calculus, faulty restorations, and dental appliances [2, 3]. It is typically seen as a gingival growth on interdental papilla and comprises about 9% of all gingival growths [1]. Females are more commonly affected, and anterior maxilla is the most prevalent location [1]. POFs are usually less than 1.5 cm in diameter, and diagnosis can be made by clinical inspection and biopsy [4]. It has not been clarified whether POF is a tumor or represents proliferation of a reactive nature. POF shows a clinically benign behavior [5]. Incidences of recurrence have been put at 16–20% [6]. The reasons for recurrence include incomplete removal of lesion, failure to eliminate local irritants, and difficulty in access during surgical manipulation due to intricate location of POF being present usually at interdental areas. Deep excisions have been preferred for recurrences [6].

## 2. Case Report 

A 60-year-old female patient reported with the chief complaint of painless growth on the gingiva in the upper left front region of mouth three years ago. It had progressed gradually to increase in size and attained the present size. Growth was associated with bleeding on brushing occasionally. The patient did not give any history of trauma.

**Figure 1:**
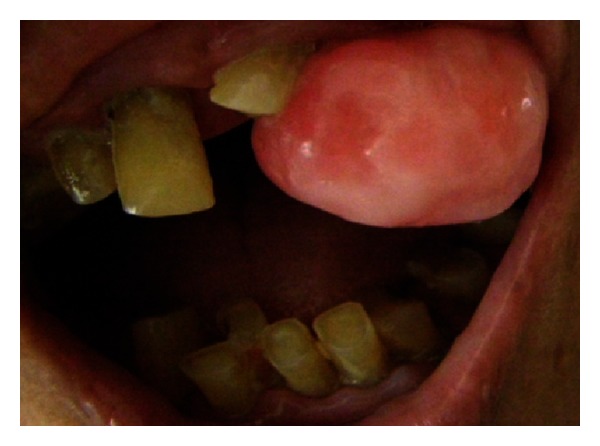


Intraoral examination revealed a solitary, sessile growth present on the residual ridge of the missing 23 in the interdental space between 22 and 24, extending mesiodistally from the distal aspect of 22 up to mesial aspect of the 24 region (Figure 1).

**Figure 2:**
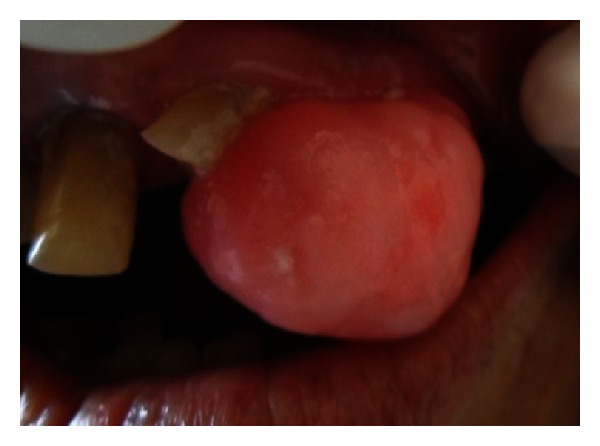


**Figure 3:**
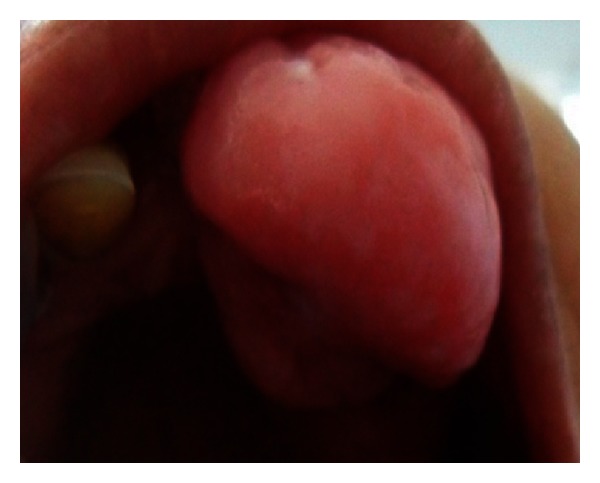


Superoinferiorly extending superiorly from marginal gingiva of 22 and 24 and inferiorly from 1-2 cm below the incisor line (Figure 2). Antero-posteriorly extending from inner aspect of upper lip to 3 cm towards hard palate (Figure 3).

It was of the same color of the adjacent gingiva with the mesial half being more reddish in color. The growth was oval in shape and approximately 2.5 × 3 cm in size in greatest dimensions with well-defined borders. The surface of the growth was lobulated. 

No secondary changes were seen related to ulceration and fungation. The growth was firm and nontender on palpation. It was nonfluctuant, nonreducible, and noncompressible with mild bleeding on probing.

The clinical differential diagnoses for the growth were pyogenic granuloma, traumatic fibroma, peripheral giant cell granuloma, and peripheral ossifying fibroma, and provisional diagnosis of pyogenic granuloma with respect to the 22, 23, and 24 regions was made for the gingival growth.

**Figure 4:**
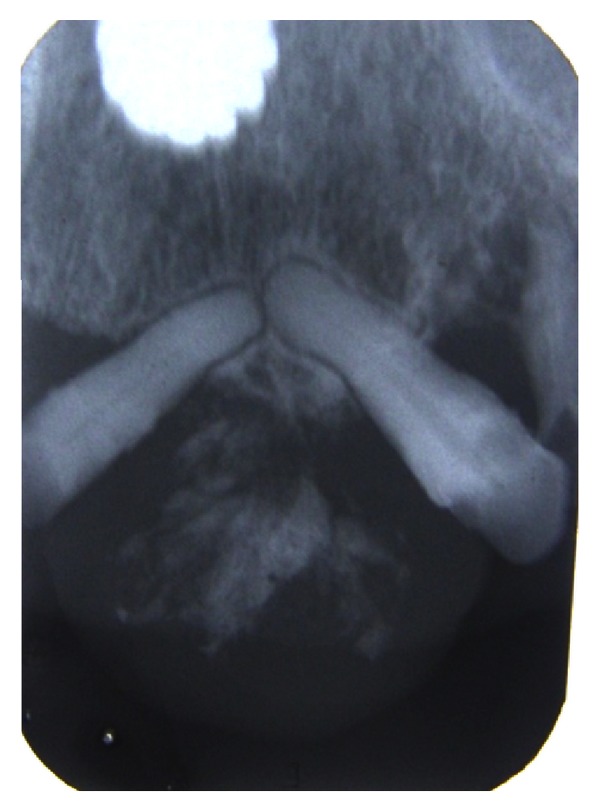


Radiological investigations—intraoral periapical radiograph of left maxillary anterior region revealed the presence of 22 and 24 and missing 23. Evidence of faint, irregular radiopacity was noticed in the missing 23 region, suggestive of soft tissue mass, and mesial tipping of 22 and distal tipping of 24 were noticed (Figure 4).

**Figure 5:**
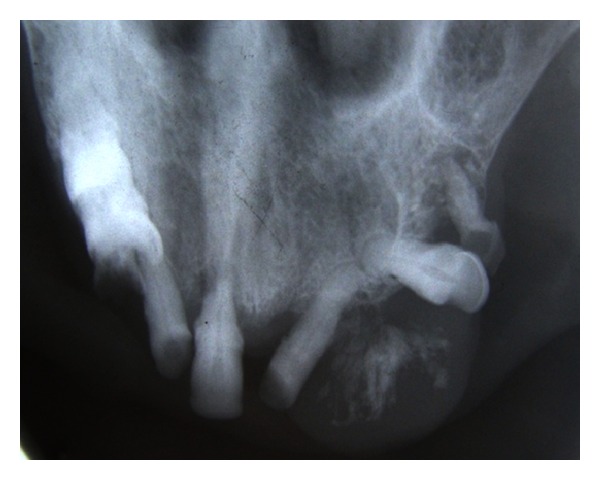


Maxillary occlusal radiograph reveals the presence of 11, 12, 22, 24, and 25. Irregular radiopacity is seen interspersed in the soft tissue shadow. The density of which is almost similar to the bone, signifying the presence of ossification (Figure 5).

## 3. Treatment

Growth was excised conservatively. The excised tissue was oval, 2.5 × 3 cm in size, pale white in color, and firm in consistency. Tissue was sent for histopathological examination. Adjacent teeth were scaled to remove the local irritants. The patient was recalled after one week for review (Figure 6).

Histologically, the specimen showed parakeratinized stratified squamous epithelium overlying the connective tissue stroma. Epithelium showed hyperplasia in some areas. Connective tissue stroma consisted of highly cellular mass of proliferating fibroblasts intermingled with fibrillar tissue. Fibrous connective tissue also consisted of large and small trabeculae of bone and some dystrophic calcifications. Based on history, clinical presentation, and radiological and histopathological examination, final diagnosis of peripheral ossifying fibroma with respect to the 22, 23, and 24 regions was put forth. 

## 4. Discussion

Menzel first described the lesion ossifying fibroma in 1872, but its terminology was given by Montgomery in 1927 [7]. Peripheral ossifying fibroma occurs mostly in craniofacial bones and categorized into two types central and peripheral. The central type of ossifying fibroma arises from the endosteum or the periodontal ligament (PDL) adjacent to the root apex and expands from the medullary cavity of the bone, and the peripheral type occurs on the soft tissues overlying the alveolar process [8]. POF is a solitary, slow growing nodular mass that is either pedunculated or sessile. Most often it is located in the gingival papilla between adjacent teeth [2].

Though the etiopathogenesis of POF is uncertain, origin from cells of periodontal ligament has been suggested. The reasons for considering periodontal ligament origin include excessive occurrence of POF in the gingival interdental papilla, the proximity of the gingival to periodontal ligament, the presence of oxytalan fibres within the mineralized matrix of some lesion, and the fibrocellular response in periodontal ligament [2, 8]. Migration of teeth with interdental bone destruction has been reported in some of the cases [9].

In vast majority of cases, there is no apparent underlying bone involvement visible on the roentgenogram. However, superficial erosion of bone is noted occasionally [2, 8, 9].

Peripheral ossifying fibroma has to be differentiated from traumatic fibroma, peripheral giant cell granuloma, pyogenic granuloma, and peripheral odontogenic fibroma.

 Peripheral odontogenic fibroma is an uncommon neoplasm that is believed to arise from odontogenic epithelial rests in periodontal ligament or attached gingiva itself. Traumatic fibroma occur on buccal mucosa along the bite line. Pyogenic granuloma presents as soft, friable nodule, small in size that bleeds with tendency to hemorrhage and may or may not occasionally or do not show calcifications but tooth displacement and resorption of alveolar bone are not observed. Peripheral giant cell granuloma has clinical features similar to POF however POF lacks the purple or blue discoloration commonly associated with peripheral giant cell granuloma and radiographically shows flecks of calcification [10].

It is possible to histologically differentiate PGCG and peripheral odontogenic fibroma from POF as PGCG contains giant cells, whereas peripheral odontogenic fibroma contains odontogenic epithelium and dysplastic dentine; all the features are not seen in POF [11].

Treatment includes local surgical excision and oral prophylaxis [12]. Followup is essential because of the recurrence rates. Recurrence is due to incomplete excision and/or due to persistence of local factors [2].

## 5. Conclusion

POF being one of the commonest solitary swelling in the oral cavity is many times clinically diagnosed as pyogenic granuloma. Radiological and histopathological examination is required for confirmation of diagnosis.

## Figures and Tables

**Figure 6:**